# Histopathological Comparison and Expression Analysis of COL1A1, COL3A1, and ELN in the Proximal and Distal Ventral Dartos of Patients With Hypospadias: Protocol for Prospective Case-Control Study

**DOI:** 10.2196/70075

**Published:** 2025-02-18

**Authors:** Putu Angga Risky Raharja, Ponco Birowo, Lisnawati Rachmadi, Heri Wibowo, Aria Kekalih, Gede Wirya Kusuma Duarsa, Tariq Abbas, Irfan Wahyudi

**Affiliations:** 1 Doctoral program in Medical Sciences Faculty of Medicine University of Indonesia Jakarta Indonesia; 2 Department of Urology, Faculty of Medicine Cipto Mangunkusumo Hospital University of Indonesia Jakarta Indonesia; 3 Department of Anatomical Pathology, Faculty of Medicine Cipto Mangunkusumo Hospital University of Indonesia Jakarta Indonesia; 4 Department of Community Medicine Faculty of Medicine University of Indonesia Jakarta Indonesia; 5 Department of Urology, Faculty of Medicine Ngoerah Hospital Udayana University Bali Indonesia; 6 Pediatric Urology Section Sidra Medicine Doha Qatar; 7 College of Medicine Qatar University Doha Qatar

**Keywords:** chordee, superficial chordee, COL1A1, COL3A1, dartos tissue, dartos fascia, ELN, elastin, histopathological

## Abstract

**Background:**

The exact cause of penile curvature in hypospadias remains unknown. Resection of the dartos fascia has been observed to straighten the penis, indicating the involvement of the dartos fascia in the superficial chordee. However, the characteristics of dartos tissue in the distal territory of the ventral penile shaft may differ from those in the proximal aspect of the penile shaft.

**Objective:**

This study aims to investigate the distinct histopathological profiles and expression of COL1A1 (collagen type 1), COL3A1 (collagen type 3), and ELN (elastin) in proximal and distal ventral dartos of patients with hypospadias compared to those without hypospadias.

**Methods:**

This prospective case-control study compares the ventral dartos tissue of patients with hypospadias at different locations with that of patients without hypospadias. Dartos samples will be taken during surgery, with age matching. Histopathology examination uses hematoxylin and eosin and Masson’s trichrome stain. The mRNA expression of *COL1A1*, *COL3A1*, and *ELN* will be quantified using a 2-step reverse transcription–polymerase chain reaction analysis.

**Results:**

Previous studies have documented different characteristics of dartos tissue between patients with hypospadias and those without hypospadias. Some studies even suggest resection of the dartos tissue during hypospadias repair. However, this is the first study to compare the characteristics of ventral dartos tissue in patients with hypospadias based on its location along the penile shaft, suggesting potential differences between the distal and proximal locations. We have obtained ethical approval to conduct a prospective case-control study aimed at elucidating these differences in dartos tissue characteristics. The findings of the study are anticipated to be available by 2025.

**Conclusions:**

Differences in the characteristics of dartos fascia based on its location may require tailored surgical strategies. If the properties of distal dartos tissue closely mirror those of typical dartos tissue, the possibility of avoiding its excision during hypospadias surgery could be considered.

**International Registered Report Identifier (IRRID):**

DERR1-10.2196/70075

## Introduction

Hypospadias is a common congenital malformation characterized by the displacement of the urethral meatus on the ventral side of the penis, incomplete ventral prepuce development, and penile curvature known as chordee [1-3]. Hypospadias represents the most prevalent form of penile deformity, with data indicating its occurrence in 1 out of every 125-300 male births [4-6]. The severity of this anomaly varies, with meatus locations ranging from the glans to the scrotum [3].

Hypospadias is typically categorized as either distal or proximal, depending on the location of the meatus [7]. Approximately 70% of cases are classified as distal hypospadias, typically considered as less severe [3,8]. However, certain variants of distal hypospadias may present with severe chordee, necessitating more intricate reconstruction procedures [9]. Chordee, one of the triads of hypospadias, can be further classified as either superficial or deep [6,10]. Superficial chordee involves the superficial fascia, whereas deep chordee affects deeper structures such as the deep fascia, urethral plate, corpus spongiosum, and tunica albuginea, and may involve a disproportion of corpora [10].

The exact cause of the chordee remains unknown. However, resection of the dartos fascia during hypospadias procedures has been observed to straighten the penis, indicating the involvement of the dartos fascia in the superficial chordee [11]. Changes in the structure of collagen (predominantly collagen type 1), reticulin (collagen type 3), and elastin components within the dartos fascia could influence its elasticity and mobility in cases of hypospadias [12]. Previous research has indicated that dartos fascia in hypospadias tends to be thicker and less elastic [13,14]. Dartos fascia also exhibited lower concentrations of collagen types 1, 2, and 3 compared to the normal control group [15].

Studies exploring the association between dartos fascia and chordee remain limited, primarily consisting of qualitative studies [16]. Some studies have suggested the resection of dartos fascia during hypospadias reconstruction surgery [15]. However, there is a lack of studies comparing the characteristics of ventral dartos in patients with hypospadias based on their location towards the meatus, classified as proximal and distal dartos. Variations in dartos fascia characteristics according to location may necessitate different surgical approaches. Therefore, this study aims to investigate the distinct histopathological profiles and expression of COL1A1 (collagen type 1), COL3A1 (collagen type 3), and ELN (elastin) in proximal and distal ventral dartos of patients with hypospadias compared to those with a normal penis.

## Methods

### Study Design

This study is a prospective case-control study comparing ventral dartos in cases of proximal and distal phenotypic variants of patients with hypospadias with dartos of patients without hypospadias. Dartos samples will be collected during hypospadias repair surgery (cases) and circumcision (controls), with the matching of ages between cases and controls and random allocation. The protocol has been developed in accordance with the 2013 SPIRIT (Standard Protocol Items: Recommendations for Interventional Trials) checklist for reporting protocol studies [17].

### Study Population and Recruitment

Patients will be recruited from our university-affiliated academic tertiary care hospitals in Jakarta, Indonesia. The inclusion criteria for the cases are as follows: (1) male patients with prepuberty from 6 months to 9 years; (2) a confirmed diagnosis of hypospadias, established through physical examination by a pediatric urologist; and (3) undergoing hypospadias repair at our hospitals during the recruitment period. Conversely, the exclusion criteria for cases are specified as follows: (1) a history of previous hypospadias repair, (2) inadequate tissue samples for analysis, and (3) undescended testis.

The inclusion criteria for the control group consist of (1) male patients with prepuberty from 6 months to 9 years, (2) confirmation of a normal penile condition through a physical examination conducted by a urologist, and (3) undergoing circumcision at our hospitals during the recruitment period. Conversely, the exclusion criteria for controls are outlined as follows: (1) a history of previous genital surgery, (2) insufficient tissue samples for analysis, and (3) undescended testis.

Participants meeting the inclusion criteria and not meeting any of the exclusion criteria will be invited to participate in the study as either cases or controls. According to the sample size formula (5% α and 20% β), each group requires approximately 50 patients (rounded up from 43.18). Considering our patient volume, we anticipate completing recruitment within a 9- to 12-month time frame.

### Data Collection

The following data will be collected:

- Patient demographics: information such as age and socioeconomic status.
- Clinical variables: detailed medical history, associated comorbidities, family history, and birth weight.
- Phenotypic data being captured: location of the urethral opening, plate objective screening tool, urethral defect ratio, penile curvature assessment, and glans-urethral meatus-shaft hypospadias score.

#### Location of Urethral Opening

**Figure 1:**
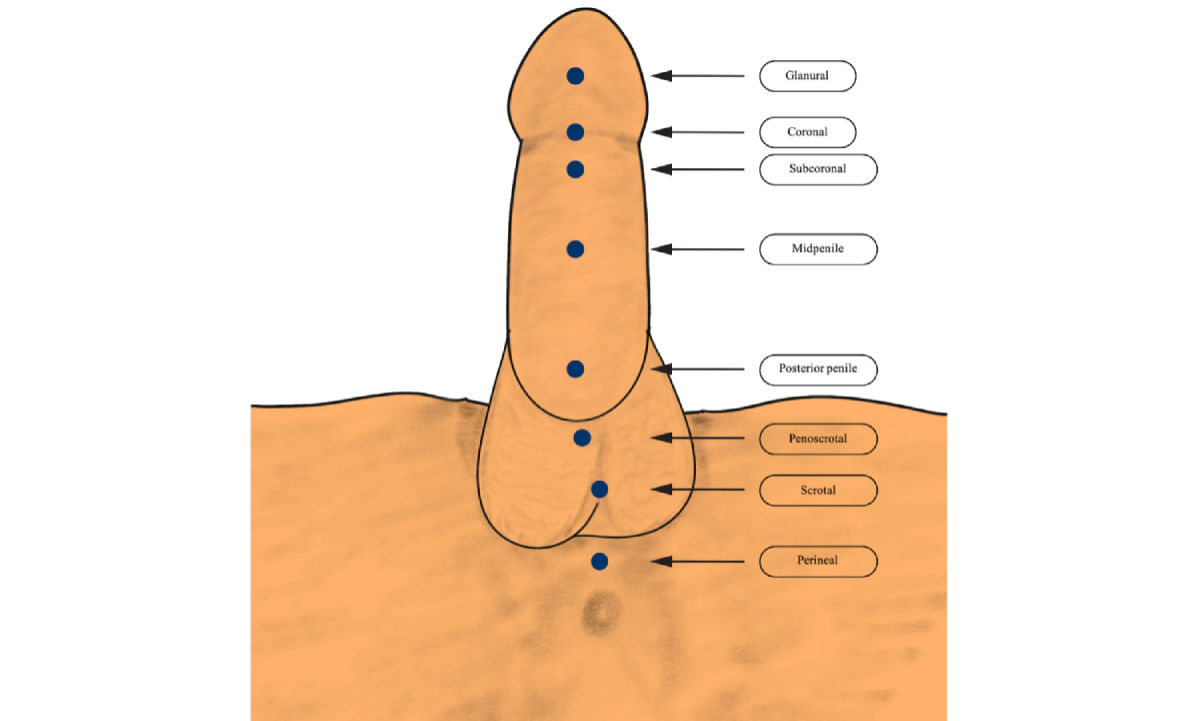
Location of the urethral opening.

The location of the urethral opening is given in Figure 1 [18].

#### Plate Objective Scoring Tool

**Figure 2:**
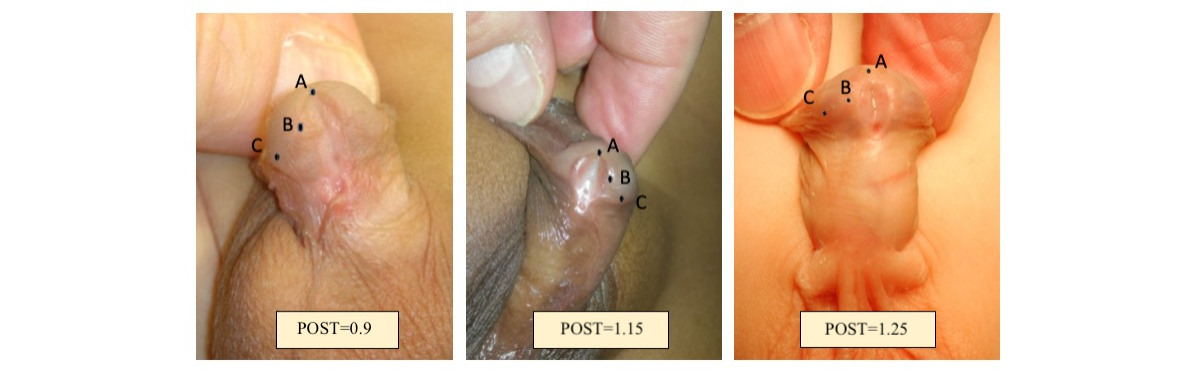
Plate objective scoring tool score calculation (AB/BC). POST: Plate objective scoring tool.

Plate objective scoring tool (POST) score is shown in Figure 2 [19,20]. The distal extent of the urethral plate at the midline is identified as point A. The glanular knob, where the mucosal edge of the plate changes direction, is marked as point B, while the glanular-coronal junction is designated as point C. The distance from point A to point B represents the neomeatal length, while the distance from point B to point C indicates the length of the prospective glanular fusion. The POST score has been used to quantify the urethral plate quality in distal hypospadias. This tool’s correlation with postrepair complications underscores its relevance in optimizing surgical outcomes [20].

#### Urethral Defect Ratio

**Figure 3:**
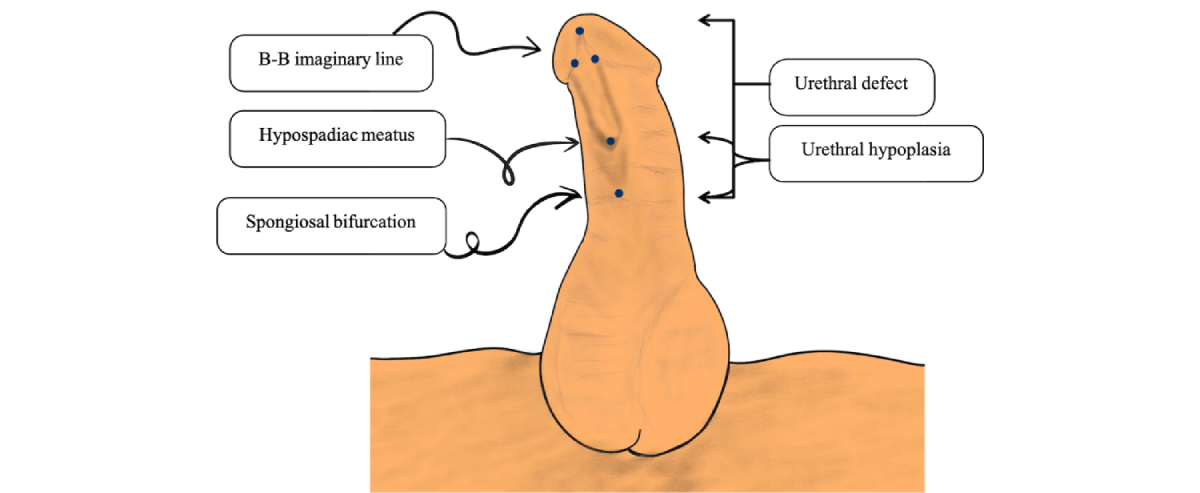
Urethral defect ratio. B–B: the distance between the glandular knobs.

Urethral defect ratio (UDR), which is illustrated in Figure 3, is an objective hypospadias classification system [21]. The stretched penile length is measured along the lateral aspect of the penile shaft, extending from the tip of the glans to the superior border of the pubic bone. Stretching is facilitated using a stay suture in conjunction with a metal ruler. The bifurcation site of the corpus spongiosum penile curvature is evaluated following complete degloving of the penile skin. The distance between the glandular knobs (B–B imaginary line) and the bifurcation site of the corpus spongiosum penile curvature is defined as the urethral defect, and the UDR is calculated by dividing UD by stretched penile length [21].

#### Penile Curvature Assessment

**Figure 4:**
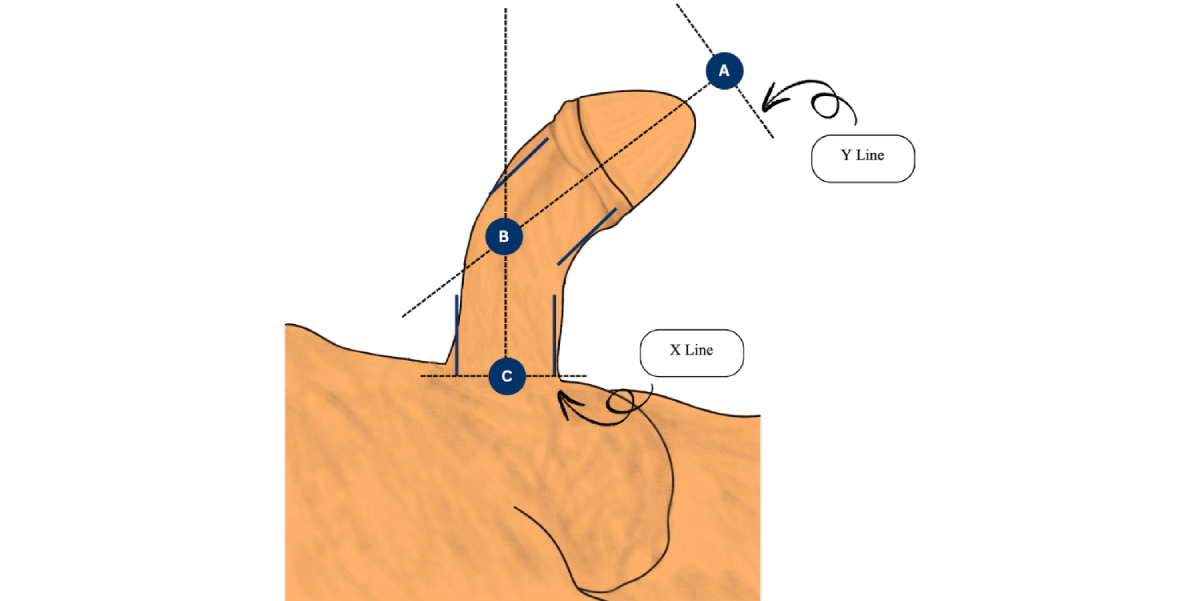
Penile curvature assessment. The distal midpoint (A); the point of angulation (B); and the penile base (C).

Penile curvature assessment is shown in Figure 4 [22]. To evaluate penile curvature intraoperatively, we have established a standardized protocol using mobile apps. This involves capturing a lateral photograph of the penis from a distance of 25-30 cm, ensuring alignment parallel to the penile shaft, and incorporating an upward oblique angle between 10° and 30° along the same vertical axis. The captured image is subsequently processed using specialized software, such as Angle Meter 360, where 3 critical anatomical landmarks are marked: the penile base, the point of angulation, and the distal midpoint.

In Figure 4, three key reference points are established for measurement: (1) corresponds to the intersection of the midline axis of the distal penile shaft with the Y-line, defined as a perpendicular line originating from the tip of the glans, (2) represents the intersection of the midline axes of the proximal and distal segments of the penile shaft, and (3) signifies the intersection of the midline axis of the proximal penile shaft with the X-line, a perpendicular line drawn at the level of the pubic bone. The degree of penile curvature is calculated by subtracting the measured angle from 180°.

Glans-Urethral Meatus-Shaft Hypospadias Score.

In the glans-urethral meatus-shaft (GMS) scoring system, each of its 3 components is assigned a numerical value ranging from 1 to 4 [23]. The total GMS score can range from a minimum of 3, indicative of very mild hypospadias, to a maximum of 12, representing severe hypospadias. Studies have demonstrated its predictive value for postoperative complications, highlighting its utility in guiding surgical decision-making [24].

#### Dartos Fascia Sampling

**Figure 5:**
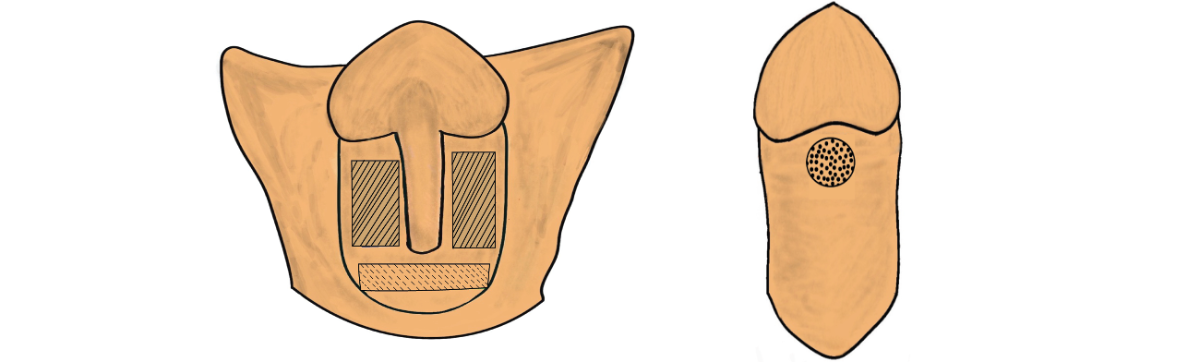
Dartos fascia sampling in the hypospadias group (solid line: distal dartos, dashed line: proximal dartos) and control group (circle).

Dartos fascia samples are obtained following penile degloving in the hypospadias group. The dartos tissue is collected from the ventral side at two distinct locations: distal to the urethral meatus (distal to spongiosa bifurcation) and proximal to the urethral meatus (proximal to spongiosa bifurcation). Conversely, in the control group, dartos tissue is acquired from the ventral side coronal area. The illustration of the samples’ location is shown in Figure 5. The size of dartos tissue collected at each location ranges from 25 to 100 mm^2^. To mitigate collection bias, all samples are obtained by a single surgeon.

#### Histopathology Preparation

Dartos samples will be immersed in a 10% buffered formaldehyde solution for fixation. Subsequently, the fixed tissue will undergo dehydration, infiltration, and embedding in liquid paraffin to achieve solidification. The paraffin-embedded blocks will then be sectioned using a microtome at a thickness of 4-5 µm and mounted onto glass slides. Two distinct staining methods, namely hematoxylin and eosin and Masson’s trichrome stain, will be used to assess the cross-sectional area and collagen percentage of the dartos tissue. All histopathological assessments and quantifications will be conducted in a blinded manner using the QuPath software.

#### Reverse Transcription–Polymerase Chain Reaction Analysis

Messenger ribonucleic acid expression levels of collagen genes (*COL1A1* and *COL3A1*) and elastin gene (*ELN*) will be quantified through a two-step reverse transcription–polymerase chain reaction analysis. Dartos tissue samples will be preserved with RNA later, followed by total RNA extraction using an RNA extraction kit. Subsequently, the extracted RNA will undergo reverse transcription to synthesize complementary deoxyribonucleic acid, which will then be amplified via PCR to detect the expression levels of the target genes.

### Statistical Analysis

All data will undergo analysis using SPSS (version 24.0; IBM Corp) software. Descriptive statistics will be used, presenting frequency for categorical variables and mean or median for numerical variables. Comparative analysis of collagen percentage and expression of *COL1A1*, *COL3A1*, and *ELN* among proximal hypospadias dartos, distal hypospadias dartos, and normal dartos will be conducted using either 1-Way ANOVA or Kruskal-Wallis test, contingent upon the normality of data distribution. Post hoc analysis may use the Bonferroni test, Games-Howell test, or Mann-Whitney test as deemed appropriate. Subanalysis based on hypospadias classification (distal and proximal) and chordee severity (mild, moderate, or severe) will also be performed. Subanalysis based on the UDR and POST scores will also be performed [17].

### Ethical Considerations

The Medical Research Ethics Committee at Universitas Indonesia approved our study protocol in March 2024 (KET-473/UN2.F1/ETIK/PPM.00.02/2024). Participants and/or their guardians will receive comprehensive information both verbally and in writing to ensure informed consent. Upon choosing to participate, they will be requested to sign the informed consent form.

## Results

Previous studies have documented different characteristics of dartos tissue between hypospadias and patients without hypospadias [11,13-15]. Some studies even suggest resection of the dartos tissue during hypospadias repair [15]. However, no previous study has systematically compared the distinct characteristics of proximal and distal dartos tissue in patients with hypospadias. We have obtained ethical approval to conduct a prospective case-control study aimed at elucidating these differences in dartos tissue characteristics. The findings of the study are anticipated to be available by 2025.

## Discussion

### Anticipated Findings

The exact etiology of chordee in hypospadias remains unknown. Nonetheless, inadequate management of chordee during hypospadias repair may escalate the incidence of complications. Moreover, significant chordee can impede sexual function in later adulthood [10]. The aberrant curvature may result in ineffective insemination, painful erections, or hindered vaginal insertion. Therefore, meticulous management of chordee in hypospadias is imperative [18].

Numerous studies have indicated abnormalities in dartos tissue in hypospadias [11,13-15]. Surgical procedures involving penile degloving and dartos tissue excision have been proposed to alleviate superficial chordee [15,18]. However, our preliminary observations during hypospadias surgery have revealed distinct characteristics between the dartos tissue located proximally and distally to the meatus. Specifically, we have noted that the proximal dartos tissue tends to exhibit greater thickness and reduced elasticity, potentially contributing to superficial chordee. Consequently, we aim to conduct a comparative analysis of the extracellular matrix properties of distal and proximal dartos tissue in this study. Variations in dartos fascia characteristics according to location may necessitate different surgical approaches. Should the characteristics of the distal dartos resemble those of normal dartos tissue, sparing its resection during hypospadias surgery may be feasible. Using the distal dartos as a secondary layer for neourethral coverage could potentially enhance surgical outcomes.

### Conclusion

Differences in the characteristics of dartos fascia based on its location may require tailored surgical strategies. If the properties of distal dartos tissue closely mirror those of typical dartos tissue, the possibility of avoiding its excision during hypospadias surgery could be considered.

## Abbreviations

COL1A1: collagen type 1
COL3A1: collagen type 3
ELN: elastin
GMS: glans-urethral meatus-shaft
POST: plate objective scoring tool
SPIRIT: Standard Protocol Items: Recommendations for Interventional Trials
UDR: urethral defect ratio

## Data Availability

The datasets generated or analyzed during this study are available from the corresponding author on reasonable request.
